# Design of Refractive Index Sensors Based on Valley Photonic Crystal Mach–Zehnder Interferometer

**DOI:** 10.3390/s25113289

**Published:** 2025-05-23

**Authors:** Yuru Li, Hongming Fei, Xin Liu, Han Lin

**Affiliations:** 1 College of Physics and Optoelectronics, Taiyuan University of Technology, Taiyuan 030024, China; 2023511408@link.tyut.edu.cn (Y.L.); liuxin@tyut.edu.cn (X.L.); 2 Shanxi Key Laboratory of Precision Measurement Physics, Taiyuan University of Technology, Taiyuan 030024, China; 3 State Key Laboratory of Quantum Optics and Quantum Optics Devices, Shanxi University, Taiyuan 030006, China; 4 Centre for Atomaterials and Nanomanufacturing, School of Science, RMIT University, Melbourne, VIC 3000, Australia; han.lin2@rmit.edu.au

**Keywords:** refractive index sensor, topological photonic crystal, valley photonic crystal, Mach–Zehnder interferometer, sensitivity

## Abstract

**Highlights:**

**What are the main findings?**

- For the first time, a valley photonic crystal Mach–Zehnder interferometry refractive index sensor with a high forward transmittance of 91% is demonstrated.
- The refractive index sensor exhibits high sensitivity, 1534%/RIU, with micrometer-scale dimensions.

**What is the implication of the main finding?**

- Valley photonic crystals can be utilized in the design of optical sensors with high transmittance and sensitivity.
- The design is suitable for various on-chip sensing applications.

**Abstract:**

The refractive index is an important optical property of materials which can be used to understand the composition of materials. Therefore, refractive index sensing plays a vital role in biological diagnosis and therapy, material analysis, (bio)chemical sensing, and environmental monitoring. Conventional optical refractive index sensors based on optical fibers and ridge waveguides have relatively large sizes of a few millimeters, making them unsuitable for on-chip integration. Photonic crystals (PCs) have been used to significantly improve the compactness of refractive index sensors for on-chip integration. However, PC structures suffer from defect-introduced strong scattering, resulting in low transmittance, particularly at sharp bends. Valley photonic crystals (VPCs) can realize defect-immune unidirectional transmission of topological edge states, effectively reducing the scattering loss and increasing the transmittance. However, optical refractive index sensors based on VPC structures have not been demonstrated. This paper proposes a refractive index sensor based on a VPC Mach–Zehnder interferometer (MZI) structure with a high forward transmittance of 0.91 and a sensitivity of 1534%/RIU at the sensing wavelength of *λ* = 1533.97 nm within the index range from 1.0 to 2.0, which is higher than most demonstrated optical refractive index sensors in the field. The sensor has an ultracompact footprint of 9.26 μm × 7.99 μm. The design can be fabricated by complementary metal–oxide semiconductor (CMOS) fabrication technologies. Therefore, it will find broad applications in biology, material science, and medical science.

## 1. Introduction

The refractive index is one of a material’s key optical properties and is closely related to its atomic structure, composition, polarizability, density, and electronic band structure. Therefore, refractive index sensing has broad applications in biomedicine [1,2,3], environmental monitoring [4], analytical chemistry [5], and material analysis [6]. An optical refractive index sensor [7,8] is a detection device that can convert changes in the refractive index into detectable optical information, which can be achieved via optical phenomena, such as resonance and interference, according to the different refractive index characteristics of different target analytes, by detecting the shift of the resonance peak or the attenuation of optical power to achieve target detection. Optical refractive index sensors have the characteristics of high-density integration, easy operation, high sensitivity, and adaptability to harsh environments [9].

At present, several types of optical refractive index sensors have been demonstrated: surface plasmon-based resonance sensors [10], fiber grating sensors [11], and interferometric sensors [12,13,14]. Plasmon-based resonance sensors [15] use the resonance effect of plasmonic waves and incident light on the surface of metal films to detect changes in the refractive index. When the refractive index of the sample to be measured varies, the propagation characteristics of the surface plasmon wave will change, resulting in a shift in the resonant angle or resonant wavelength, which can be accurately measured [16]. Fiber grating sensors show the shift in the reflection or transmission peaks resulting from the refractive index in the external environment, which allows for long-range detection [11,17]. In comparison, interferometric sensors obtain the refractive index of a substance by detecting the shift in the interference peaks or valleys due to the phase change introduced by the refractive index change [18]. Among those refractive index sensing mechanisms, interferometric sensors have high sensing accuracy and can detect very subtle refractive index changes. In addition, compared to plasmonic sensors, interferometric sensors can be realized busingy simple waveguide structures without sophisticated plasmonic structures. On the other hand, compared to the large size of fiber grating sensors, interferometric sensors can have a much smaller size, thus making them suitable for on-chip integration.

Common interferometric sensors include Mach–Zehnder interferometer (MZI) sensors [19], Michelson interferometric sensors [20], Fabry–Perot sensors [21], etc. Among the different types of interferometric sensors, MZI sensors have a simple structure, high sensitivity, and a tunable sensing range, making them a preferred solution in refractive index sensing [22,23,24]. Initially, traditional MZI refractive index sensors are composed of optical fibers, which are bulky (typically hundreds of microns in cross-section and meters in length) and unsuitable for on-chip integration. The recent development of MZIs based on waveguide structures (hundreds of nanometers in cross-section) significantly improves the compactness of MZI sensors, allowing fpr on-chip integration, which, however, still requires a millimeter footprint. In comparison, MZIs based on photonic crystal (PC) structures [25,26] can further improve the compactness (down to the micron scale) due to the large effective refractive index of the propagation mode in the PC waveguide. However, the defects could introduce strong scattering in PC MZI refractive index sensors, compromising the transmittance [27].

Topological photonic crystals (TPCs) [28,29,30] have edge states to achieve defect-immune unidirectional transmission, thus minimizing the scattering loss due to the defects at the sharp corners. Among different TPC designs, valley photonic crystals (VPCs) [31,32,33,34,35,36] can be constructed by breaking the spatial reversal symmetry without requiring an external magnetic field [37,38]. In the edge states of VPCs, there exists a spin–valley locking effect which can be used to control the propagation path of optical waves and suppress inter-valley scattering, leading to defect-immune unidirectional transmission [39]. As a result, VPCs working in the telecommunication wavelength range have been demonstrated experimentally, confirming the broad applications [40,41]. Therefore, a refractive index sensor based on a VPC MZI structure could have great potential; however, it has not been demonstrated.

Here, we demonstrate a refractive index sensor based on a VPC MZI structure, which has a high forward transmittance of 0.91 and a sensitivity of 1534%/RIU (λ = 1533.97 nm) within the index range from 1.0 to 2.0. The sensor has a small footprint of 9.26 μm × 7.99 μm. The design can be fabricated by complementary metal–oxide semiconductor (CMOS) fabrication technologies. Therefore, it will find broad applications in biology, material science, medical science, and environmental monitoring. In addition, the working principle can be broadly applied to construct devices in the fields of telecommunications [42,43] and quantum computing [44,45].

## 2. The Design of the Valley Photonic Crystal

**Figure 1:**
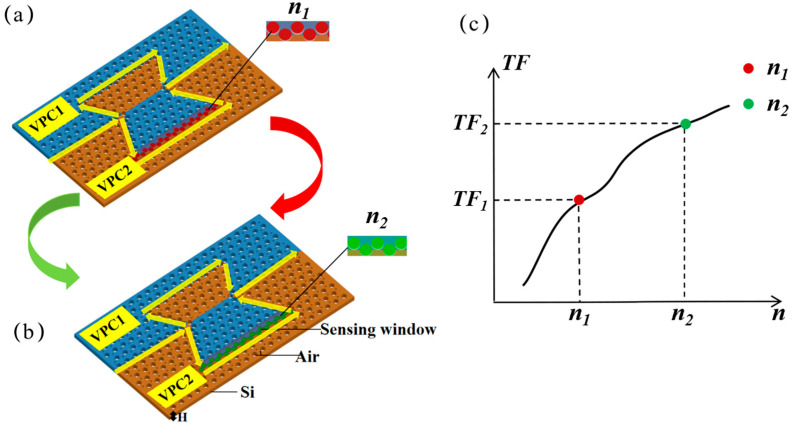
A schematic of the VPC MZI structure for refractive index sensing, where the green and red parts of the sensing arm in the MZI represent low (**a**) and high (**b**) refractive indices, respectively. The yellow arrow indicates the light path in the MZI sensors. H is the thickness of the silicon substrate. (**c**) A schematic diagram of the change in transmittance with the change in the refractive index at wavelength *λ*, where the red and green circles mark the forward transmittance *TF*_1_ and *TF*_2_ corresponding to the refractive indices of *n*_1_ and *n*_2_, respectively.

The concept of the VPC MZI refractive index sensor is shown in Figure 1, where the VPC structures are composed of holes (*r* = 80 nm) embedded in a silicon substrate with a lattice constant of *a* = 440 nm and the MZI structure consists of two straight and two Ω-shaped topological waveguides. Media with different refractive indices (*n*_1_ and *n*_2_) are injected in the sensing window (Figure 1a,b). When the refractive index increases from *n*_1_ to *n*_2_, the overall transmittance of the MZI changes continuously in the range of *TF*_1_ to *TF*_2_ at the working wavelength *λ*, as shown in Figure 1c. In this way, the refractive index of the medium can be measured according to the transmittance at *λ*.

In order to design the refractive index sensor, we first create a honeycomb lattice with C6V rotational symmetry with a Dirac point in the photonic band structure (Supplementary Figure S1). By changing the radius of the air holes, valley photonic crystals VPC1 and VPC2 are obtained, which are reduced to C3 symmetry. The K and K’ points degenerate and form a photonic bandgap (Supplementary Figure S1). Since VPC1 and VPC2 are mirror symmetric, the photonic band diagrams of VPC1 and VPC2 are the same (details of the design process are shown in Supplementary Materials, Section S1). VPC1 and VPC2 are combined to construct two possible boundary types to support the topological edge states, namely the zigzag-type and beard-type. By analyzing the edge state diagrams and transmission spectra of the topological waveguides based on the two boundaries, it is concluded that the zigzag-type waveguide has a broader working bandwidth and higher transmittance (detail are shown in Supplementary Materials, Section S2). Therefore, the zigzag-type waveguide is chosen to construct the MZI sensor. The photonic band diagram and the transmission spectra are calculated using commercial software (Lumerical FDTD2020) based on the finite difference time domain (FDTD) method.

We study the influence of different refractive indices on the edge states of the zigzag-type boundary. The refractive index *n* is selected in the range of 1.0–2.0 in this study, because this range enables the sensor to be applied in various measurement scenarios, such as biological sample detection, chemical analysis, environmental monitoring, etc. It is found that the edge states redshift as the refractive index increases (details are shown in Supplementary Materials, Section S3). Correspondingly, the working bandwidth of the topological waveguide based on the edge state redshifts as the refractive index increases (details are shown in Supplementary Materials, Section S4).

## 3. The Design of the MZI Structure

**Figure 2:**
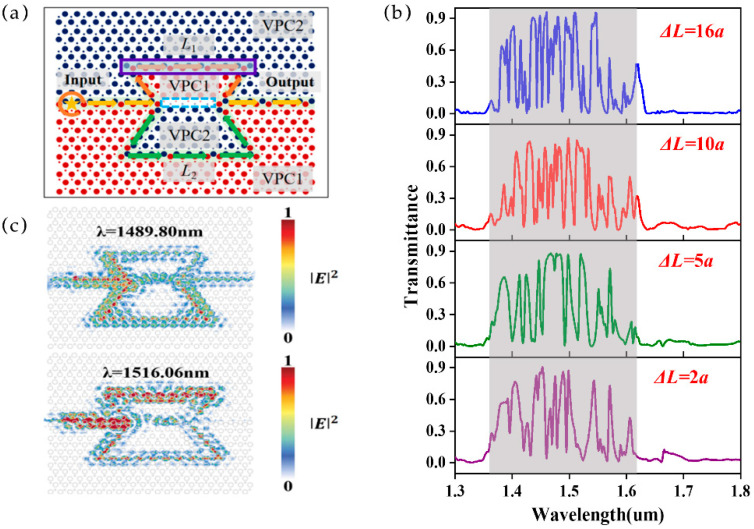
(**a**) A schematic diagram of the VPC MZI structure (Δ*L* = 5a). (**b**) The interference spectra corresponding to the MZI structure with Δ*L* = 2*a*, 5*a*, 10*a*, and 16*a*, where the gray shading region represents the working bandwidth. (**c**) The electric field intensity distributions in the MZI at 1489.80 nm and 1516.09 nm when Δ*L* is 5*a*.

The MZI structure consists of two straight topological waveguides and two interference arms, namely *L*_1_ and *L*_2_, respectively, represented by the orange and green arrows in Figure 2a. The purple box on *L*_1_ is set as the sensing region, where the test samples of different refractive indices are applied to tune the effective refractive index of *L*_1_. There is a straight waveguide composed of small holes in the middle of the MZI structure, as shown by the blue dashed line box in Figure 2a, which does not support the same edge states (details are shown in Supplementary Materials, Section S6) and thus will not affect the overall transmittance of the MZI structure.

The phase difference between the two arms of an MZI can be expressed as

(1) $$∆\varphi =\frac{2\pi }{\lambda }\cdot \left(n_{eff1}\cdot L_{1}-n_{eff2}\cdot L_{2}\right),$$

where *n_eff_*_1_ and *n_eff_*_2_ are the effective refractive indices of the sensing arm and the reference arm, and *L*_1_ and *L*_2_ are the lengths of the sensing arm and the reference arm, respectively. A π phase difference is usually required between the arms to achieve destructive interference and to obtain a near-zero valley at the designed wavelength.

The light intensity at the output can be expressed as

(2) $$I_{0}=I_{1}+I_{2}+\sqrt{I_{1}\cdot I_{2}}\cdot \cos∆\varphi ,$$

where *I*_1_ and *I*_2_ represent the transmitted light intensity in the interference arms *L*_1_ and *L*_2_, respectively, and ∆*φ* is the phase difference shown in Equation (1).

The incident light first passes through a straight waveguide with a length of 8*a* and is split into two parts (50:50), passing through the interference arms *L*_1_ (*L*_1_ = 18*a*) and *L*_2_ (*L*_2_ = 23*a*) (Figure 2a), respectively. Finally, the light from *L*_1_ and *L*_2_ interferes at the intersection. Meanwhile, the position of the peaks (constructive interference) and valleys (destructive interference) in the interference spectrum can be well controlled by designing the optical path length difference to control the phase difference between the light from the two arms. The transmission spectra of MZI refractive index sensors with different optical path length differences (Δ*L*) are shown in Figure 2b, which shows the typical interference intensity patterns of MZI structures controlled by Δ*L*. Due to the high forward transmittance in the wavelength range of 1.3–1.8 μm (marked by the gray shading in Figure 2b), the MZI shows distinct interference peaks (close to 1) and valleys (close to 0) (Figure 2b). The intensity distribution of an MZI with Δ*L* = 5*a* (*L*_1_ = 18*a* and *L*_2_ = 23*a*) at the interference peak (1489.80 nm) and valley (1516.06 nm) is shown in Figure 2c. One can see obvious constructive interference at 1489.80 nm, resulting in a high field intensity in the output waveguide. In comparison, there is almost no intensity in the output waveguide at 1516.06 nm due to the destructive interference.

We further studied the influence of the optical path length difference (Δ*L*) on the interference spectrum, which is controlled by tuning the length of *L*_1_ with *L*_2_ to remain constant. The optical path length difference Δ*L* of 2*a*, 5*a*, 10*a*, and 16*a* was studied, and we can see in Figure 2b that the number of interference peaks and valleys increases as the arm length difference increases

## 4. Refractive Index Sensing Using the MZI Structure

**Figure 3:**
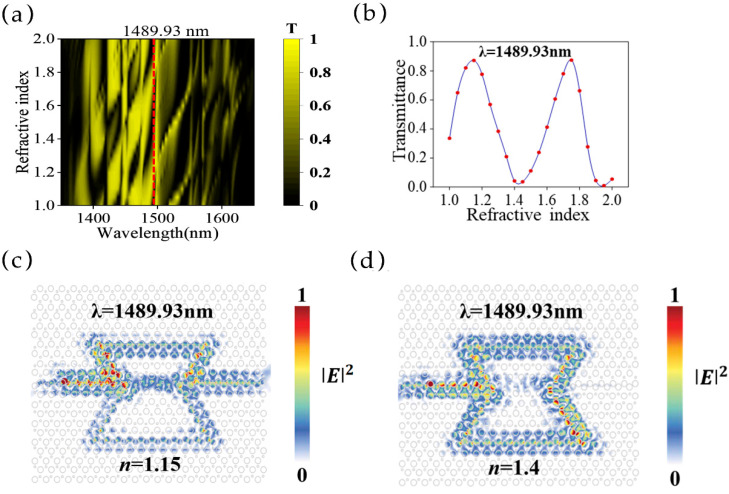
(**a**) The transmittance contour map of an MZI refractive index sensor (*L*_1_ = 18*a* and *L*_2_ = 23*a*) with different refractive indices at different wavelengths. The dashed line marked the transmittance of a single wavelength, which is plotted in (**b**). (**b**) A plot of transmittance as a function of the refractive index in the MZI at a wavelength of 1489.93 nm as marked by the dashed line in (**a**). (**c**) The electric field intensity distribution in the MZI at 1489.93 nm when *n* = 1.15. (**d**) The electric field intensity distribution in the MZI at 1489.93 nm when *n* = 1.4.

We studied a substance with a refractive index range of 1.0–2.0 and with the sensing window filled with an index step of 0.05. Here, the contour map of the intensity distributions versus the wavelength and the refractive index of an MZI structure of *L*_1_ = 18*a* and *L*_2_ = 23*a* is shown in Figure 3a, which shows distinct changes in the refractive index variation. Then, we studied the refractive index-induced intensity modulation at a single wavelength (*λ* = 1489.93 nm, marked by the red dashed line in Figure 3a), which is shown in Figure 3b. There is a semi-periodic change (similar to a sine curve) from peak to valley in the transmittance with an increased refractive index. Constructive interference happens when the refractive index is 1.15, resulting in a high forward transmittance of 0.87, and the electric field distribution is shown in Figure 3c. In comparison, destructive interference occurs when the refractive index is 1.40, resulting in a low transmittance of 0.04, and the electric field intensity distribution is shown in Figure 3d. The sensitivity defined by the change in transmittance (Δ*T*) divided by the change in the refractive index (Δ*n*), as ∆*T*⁄∆*n*, is 332%/RIU, where RIU is the refractive index unit (RIU), and the modulation depth reaches 83%.

**Figure 4:**
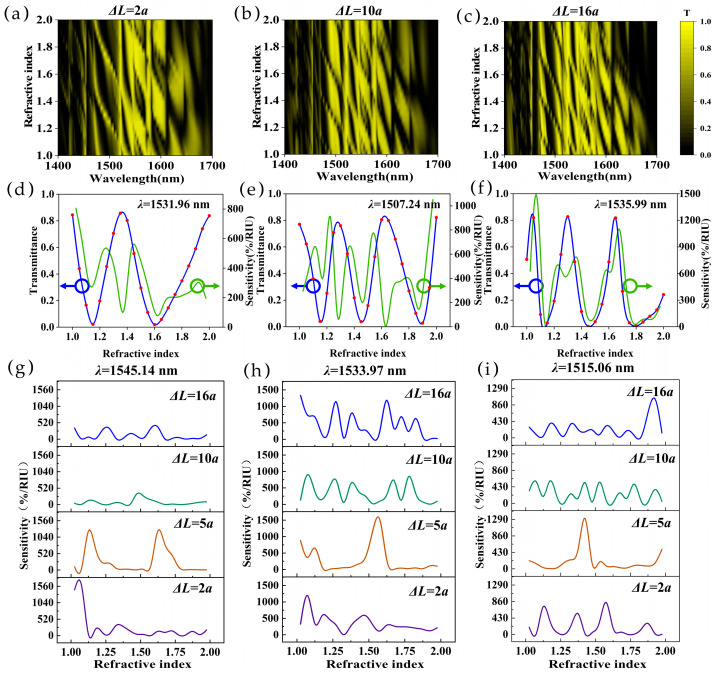
(**a**–**c**) The transmittance contour maps of the MZI with different refractive indices at different wavelengths based on MZI refractive index sensors when Δ*L* is 2*a*, 10*a*, and 16*a*. (**d**–**f**) Transmittance plots (blue spline) and sensitivity plots (green spline) as a function of refractive index in the MZI when Δ*L* is 2*a*, 10*a*, and 16*a* at wavelengths 1531.96 nm, 1507.24 nm, and 1535.99 nm, respectively. (**g**–**i**) Plots of sensitivity of the MZI structures with Δ*L* of 2*a*, 5*a*, 10*a*, and 16*a* at wavelengths of *λ* = 1545.14 nm, 1533.97 nm, and 1515.06 nm, respectively.

We further studied the path length difference Δ*L* based on the performance of refractive index sensing, in which a length difference of Δ*L* = 2*a*, 10*a*, and 16*a* was chosen in addition to the Δ*L* = 5*a* design. The resulting contour maps of the transmittance versus the refractive index and wavelength are shown in Figure 4a–c. One can see that the number of peaks and valleys increases with the increase in the path length difference Δ*L*, which is expected from the equation $∆\varphi =\frac{2\pi }{\lambda }\cdot ∆L\cdot n$, and the wavelengths of the peaks and valleys meet the conditions of ∆φ=2π·m (peak) and ∆φ=π·(2m+1) (valley), respectively, where m is an integer. Then, we studied the effect of refractive index change on the transmittance of a particular wavelength. Due to the different interference spectra, we chose different wavelengths for each length difference. Through analysis and verification, we chose 1531.96 nm (Δ*L* = 2*a*), 1507.24 nm (Δ*L* = 10*a*), and 1535.99 nm (Δ*L* = 16*a*), as shown in Figure 4d–f, which shows periodical patterns with distinct peaks and valleys. With the increase in the arm length difference, the number of peaks and valleys increases, resulting in a change in sensitivity. The sensitivity changes continuously with the increase in the refractive index, and the sensitivity tends to increase with the increase in the arm length difference.

We further analyzed the influence of specific wavelength and arm length differences on refractive index sensitivity. Three wavelengths (1545.14 nm, 1533.97 nm, and 1515.06 nm) were calculated as examples, as shown in Figure 4g–i. One can see that the sensitivity varies significantly due to the change in Δ*L* and wavelength, as well as changes in the sensing region. One example of high sensitivity can be achieved when *λ* = 1533.97 nm and Δ*L* = 5*a*; the sensitivity in the refractive index range of 1.55–1.60 is high, up to 1534%/RIU. Meanwhile, high sensitivity (>1000%/RIU) can also be achieved at wavelengths of 1545.14 nm and 1515.06 nm, as shown in Figure 4g,i, confirming the capability of VPC MZI refractive index sensors. In practical applications, the sensor spectra may be affected by environmental factors, such as temperature. Therefore, we present a detailed study on sensor responses to environmental temperature, which is provided in the Supplementary Materials, Section S8. The result demonstrates that small temperature perturbations exhibit negligible impact on the transmission characteristics, highlighting the robust thermal stability of the proposed topological photonic configuration.

Table 1 shows the performance of this VPC MZI compared to other typical MZIs. As demonstrated, the VPC MZI refractive index sensor developed in this work achieves a high sensitivity of 159 dB/RIU. Here, we compare the sensitivity with several other types of MZI refractive index sensors, including a hollow hybrid plasmonic (HP) waveguide MZI, double-slot hybrid plasmonic (DSHP) MZI, photonic crystal fiber (PCF)–single-mode fiber (SMF) MZI, slotted photonic crystal waveguide (S-PhCW) MZI, and thin-core fiber (TCF)–single-mode fiber (SMF)–thin-core fiber (TCF)-MZI (189 dB/RIU). The original sensitivity is shown in Table 1, and for a fair comparison, we have translated those sensitivities of different units into the same unit, dB/RIU. As one can see in Table 1, our design shows a high sensitivity comparable to the highest among various types of MZI refractive index sensors. Meanwhile, we also list the footprint of those MZI refractive sensors for comparison. In terms of size, our sensor features a significantly smaller footprint (9.26 × 7.99 μm^2^) compared to other structures. This highlights that the VPC MZI achieves an ultracompact design while maintaining relatively high sensitivity, demonstrating the potential of VPCs in advancing miniaturized and high-performance refractive index sensors. In addition, the designed device can be experimentally fabricated using CMOS nanofabrication technology. The CMOS compatibility and specific process steps are discussed in Supplementary Materials, Section S9. Furthermore, we present a discussion on the manufacturing tolerances of the hole radius and lattice constant in VPC design in the Supplementary Materials, Section S10, allowing us to define the required fabrication accuracy. The resulting transmittance spectrum remains largely unchanged, despite errors in the hole radius and lattice constant. The corresponding potential sources of experimental error in transmittance measurements are discussed in the Supplementary Materials, Section S11.

## 5. Conclusions

We demonstrate the fabrication of ultracompact optical refractive index sensors based on VPC MZI structures which show high sensitivity and high transmittance with small footprints at the micron scale. We further present that the arm length difference can be used to tune the sensing wavelength and the sensitivity, which provides an effective means to flexibly design a VPC MZI working at a required wavelength with a desired sensitivity. The highest sensitivity within our sensing range (1.0–2.0) is 1534%/RIU, achieved with an arm length difference of Δ*L* = 5*a* at a wavelength of *λ* = 1533.97 nm. The designed device can be experimentally fabricated using CMOS nanofabrication technology. Thus, in addition to refractive index sensing, it will also be widely used in the fields of quantum computing, optical sensing, and integrated nanophotonic devices, providing the possibility of designing more high-performance sensor devices using topological photonic crystals in the future.

## Figures and Tables

**Table 1:** Comparison of refractive index sensors based on MZI structures.

Materials	Structures	Sensitivity (For Comparison, Unify the Unit)	Footprint (Size)	Reference
SOI waveguide	HP-MZI	7.56 dB/RIU (160 nm/RIU)	20 μm	[46]
SOI waveguide	DSHP-MZI	2 dB/RIU (1061 nm/RIU)	40 μm	[47]
Photonic crystal	S-PhCW-MZI	187 dB/RIU (2.3 × 109 nm/RIU)	1 mm	[48]
Fiber	TCF-SMF-TCF MZI	189 dB/RIU (159 nm/RIU)	5 mm	[49]
Photonic crystal fiber	PCF-SMFs MZI	120 dB/RIU (198.77 nm/RIU)	3.2 cm	[50]
Silicon	VPC MZI	159 dB/RIU (1534%/RIU)	9.26 × 7.99 μm^2^	This work

## Data Availability

The data that support the findings of this study are available from the corresponding author upon reasonable request.

## Supplementary Materials

The following supporting information can be downloaded at: https://www.mdpi.com/article/10.3390/s25113289/s1. Supplementary Materials: Design of refractive index sensors based on valley photonic crystal Mach–Zehnder interferometer; Section S1: Design of Mach–-Zehnder interferometer based on valley photonic crystal; Section S2: Topological waveguide analysis with different boundary types; Section S3: Influence of different refractive indices on edge states; Section S4: Straight waveguide at different sensing refractive indices; Section S5: Omega (Ω)-shaped waveguide at different sensing refractive indices; Section S6: Topological waveguide analysis consisting of small holes; Section S7: Transmittance spectra of MZI refractive index sensors with different refractive indices; Section S8: The response of the sensor to environmental temperature fluctuations; Section S9: The CMOS compatibility statement and specific process steps; Section S10: The fabrication tolerances for the hole radius and lattice constant in the VPC design; Section S11: The potential sources of experimental error in transmittance measurements.

## References

[1] Xie M.Z., Zhang Y., Fu W.L., He J.C. (2021). Microfludic Refractive Index Sensor Based on Terahertz Metamaterials. Spectrosc. Spect. Anal..

[2] Yan D.X., Li J.S., Wang Y. (2019). High sensitivity terahertz refractive index sensor based on sunflower-shaped circular photonic crystal. Acta Phys. Sin..

[3] Li W., Long Y., Yan Y., Xiao K., Wang Z., Zheng D., Leal-Junior A., Kumar S., Ortega B., Marques C. (2025). Wearable photonic smart wristband for cardiorespiratory function assessment and biometric identification. Opto-Electron. Adv..

[4] Alsharari M., Wekalao J., Patel S.K., Kumar U.A., Aliqab K., Armghan A. (2024). Enhanced Sensing Efficiency of Ultra-Narrow Band Graphene-Based Surface lasmon Resonance Refractive Index Sensor for Biochemical Applications and Environmental Monitoring. Plasmonics.

[5] Sequeira F., Duarte D., Bilro L., Rudnitskaya A., Pesavento M., Zeni L., Cennamo N. (2016). Refractive Index Sensing with D-Shaped Plastic Optical Fibers for Chemical and Biochemical Applications. Sensors.

[6] Tsigara A., Athanasekos L., Manasis J., Hands M., Mousdis G., Pispas S., Vainos N.A. Inorganic and hybrid polymer-inorganic nanostructured materials, for optical physicochemical sensing applications. Proceedings of the ROMOPTO 2006: Eighth Conference on Optics.

[7] Liang R.B., Sun Q.Z., Wo J.H., Liu D.M. (2011). Theoretical investigation on refractive index sensor based on Bragg grating in micro/nanofiber. Acta Phys. Sin..

[8] Liao J., Feng W.L. (2022). Compatibility defects of the fiber-optic liquid level and refractive index sensors based on modal interference. Phys. B.

[9] Yuan D., Dong Y., Liu Y., Li T. (2015). Mach-Zehnder Interferometer Biochemical Sensor Based on Silicon-on-Insulator Rib Waveguide with Large Cross Section Sensors. Sensors.

[10] Zhao Y., Tong R.J., Xia F., Peng Y. (2019). Current status of optical fiber biosensor based on surface plasmon resonance. Biosens. Bioelectron..

[11] Baldini F., Brenci M., Chiavaioli F., Giannetti A., Trono C. (2012). Optical fibre gratings as tools for chemical and biochemical sensing. Anal. Bioanal. Chem..

[12] Wang R., Tang T.T., Shen J., Li C.Y. (2019). Refractive index sensing based on Mach-Zehnder interferometer with a hybrid silica/polymer waveguide. Superlatt. Microstruct..

[13] Jiang L.Z., Wu J.Y., Li Q., Deng G.W., Zhang X.L., Li Z.H., Chen K.X., Chiang K.S. (2019). A photochromic dye doped polymeric Mach-Zehnder interferometer for UV light detection. J. Mater. Chem. C.

[14] Luff B.J., Wilkinson J.S., Piehler J., Hollenbach U., Ingenhoff J., Fabricius N. (1998). Integrated Optical Mach-Zehnder Biosensor. J. Light. Technol..

[15] Miyazaki C.M., Shimizu F.M., Ferreira M. (2017). Surface Plasmon Resonance (SPR) for Sensors and Biosensors. Nanocharact. Tech..

[16] Phiri I.K., Zekriti M. (2024). Design and theoretical analysis of SPR biosensors based on gold-silver alloy and protective top layer for enhanced biosensing applications. Phys. B.

[17] Zhong X., Xie Q., Liu Y., He Y., Zhong N., Zhang Z., Karimi-Maleh H., Peng X., Lichtfouse E. (2024). Recent advances in optical fiber grating sensors for detection of organic substances. Chem. Eng. J..

[18] Tong R., Zhao Y., Hu H., Qu J. (2020). Large measurement range and high sensitivity temperature sensor with FBG cascaded Mach-Zehnder interferometer. Opt. Laser Technol..

[19] Li B.Y., Jiang L., Wang S.M., Zhou L.Y., Xiao H., Tsai H.L. (2011). Ultra-Abrupt Tapered Fiber Mach-Zehnder Interferometer Sensors. Sensors.

[20] Kaiyue Q., Yundong Z., Jianfeng S., Guo Y. (2020). All-Fiber high temperature and refractive index sensor based on three microspheres array Michelson interferometer. Opt. Laser Technol..

[21] Wang P., Pan Y., Zhang J., Zhai J., Liu D., Lu P. (2024). Miniaturized and highly sensitive fiber-optic Fabry–Perot sensor for mHz infrasound detection. Photon. Res..

[22] Guan X., Wang X., Frandsen L.H. (2016). Optical temperature sensor with enhanced sensitivity by employing hybrid waveguides in a silicon Mach-Zehnder interferometer. Opt. Express.

[23] Nohoji A.H.A., Danaie M. (2022). Highly sensitive refractive index sensor based on photonic crystal ring resonators nested in a Mach-Zehnder interferometer. Opt. Quantum Electron..

[24] Sepúlveda B., del Río J.S., Moreno M., Blanco F.J., Mayora K., Domínguez C., Lechuga L.M. (2006). Optical biosensor microsystems based on the integration of highly sensitive Mach-Zehnder interferometer devices. J. Opt. A Pure Appl. Opt..

[25] Krauss T.F., De La Rue R.M. (1999). Photonic crystals in the optical regime—past, present and future. Prog. Quantum Electron..

[26] Noda S., Chutinan A., Imada M. (2000). Trapping and emission of photons by a single defect in a photonic bandgap structure. Nature.

[27] Beggs D.M., O’Faolain L., Krauss T.F. (2009). Accurate determination of hole sizes in photonic crystal slabs using an optical measurement. Phys. E.

[28] Wang X.R., Fei H.M., Lin H., Wu M., Kang L.J., Zhang M.D., Liu X., Yang Y.B., Xiao L.T. (2023). High-performance chiral all-optical OR logic gate based on topological edge states of valley photonic crystal. Chin. Phys. B.

[29] Wu M., Yang Y.B., Fei H.M., Lin H., Han Y.H., Zhao X.D., Chen Z.H. (2022). Unidirectional transmission of visible region topological edge states in hexagonal boron nitride valley photonic crystals. Opt. Express.

[30] Wu M., Yang Y.B., Fei H.M., Lin H., Zhao X.D., Kang L.J., Xiao L.T. (2022). On-Chip Ultra-Compact Hexagonal Boron Nitride Topological Ring-Resonator in Visible Region. J. Light. Technol..

[31] Bai J., Fei H., Lin H., Wang Y., Zhang M., Liu X., Cao B., Tian Y., Xiao L. (2024). Design of a temperature sensor based on a valley photonic crystal Mach–Zehnder interferometer. Appl. Opt..

[32] Han Y.H., Fei H.M., Lin H., Zhang Y.M., Zhang M.D., Yang Y.B. (2021). Design of broadband all-dielectric valley photonic crystals at telecommunication wavelength. Opt. Commun..

[33] Kang L.J., Fei H.M., Lin H., Wu M., Wang X.R., Zhang M.D., Liu X., Sun F., Chen Z.H. (2023). Thermal tunable silicon valley photonic crystal ring resonators at the telecommunication wavelength. Opt. Express.

[34] Wang X.R., Han Y.H., Fei H.M., Lin H., Zhang M.D., Liu X., Cao B.Z., Yang Y.B., Chen Z.H., Xiao L.T. (2023). Design of wavelength division multiplexing devices based on tunable edge states of valley photonic crystals. Opt. Express.

[35] Wang Y., Fei H., Lin H., Bai J., Zhang M., Liu X., Cao B., Tian Y., Xiao L. (2024). Ultra-compact electro-optic phase modulator based on a lithium niobate topological slow light waveguide. Opt. Express.

[36] Ya M.Z., Hong M.F.E.I., Han L.I.N., Han Y.-H., Ming D.Z., Xue M.L.I., Yi B.Y. (2021). Design of all-dielectric valley photonic crystals with low symmetry elliptical lattice. J. Infrared Millim. Terahertz Waves.

[37] Cheng Q., Wang S.T., Lv J.T., Liu N. (2022). Topological photonic crystal biosensor with valley edge modes based on a silicon-on-insulator slab. Opt. Express.

[38] Peng Y.G., Geng Z.G., Zhu X.F. (2018). Topologically protected bound states in one-dimensional Floquet acoustic waveguide systems. J. Appl. Phys..

[39] Dong J., Chen X., Zhu H. (2017). Valley photonic crystals for control of spin and topology. Nat. Mater..

[40] Shalaev M.I., Walasik W., Tsukernik A., Xu Y., Litchinitser N.M. (2019). Robust topologically protected transport in photonic crystals at telecommunication wavelengths. Nat. Nanotechnol..

[41] He X.-T., Liang E.-T., Yuan J.-J., Qiu H.-Y., Chen X.-D., Zhao F.-L., Dong J.-W. (2019). A silicon-on-insulator slab for topological valley transport. Nat. Commun..

[42] Guan W., Wu Y., Xie C., Fang L., Liu X., Chen Y. (2018). Performance analysis and enhancement for visible light communication using CMOS sensors. Opt. Commun..

[43] Zhi J.L., Gui H., Cheng F.Q., You Y.F., Hong Y.W., Zhao J.L. (2021). CMOS monolithic photodetector with a built-in 2-dimensional light direction sensor for laser diode based underwater wireless optical communications. Opt. Express.

[44] Jiang D., Yu D.-Y., Zheng Z., Cao X.-C., Lin Q., Liu W.-M. (2022). Research progress of material, physics, and device of topological superconductors for quantum computing. Acta Phys. Sin..

[45] Ioudashkin E., Malka D. (2025). High-Performance O-Band Angled Multimode Interference Splitter with Buried Silicon Nitride Waveguide for Advanced Data Center Optical Networks. Photonics.

[46] Sun X., Thylén L., Wosinski L. (2017). Hollow hybrid plasmonic Mach–Zehnder sensor. Opt. Lett..

[47] Sun X., Dai D., Thylén L., Wosinski L. (2015). High-sensitivity liquid refractive-index sensor based on a Mach-Zehnder interferometer with a double-slot hybrid plasmonic waveguide. Opt. Express.

[48] Zhang Y.-N., Zhao Y., Wang Q., Xue K. (2013). Liquid refractive index sensor based on slow light in slotted photonic crystal waveguide. Optik.

[49] Gao P.A., Rong Q.Z., Sun H., Hu M.L. (2013). High-sensitive fiber-optic refractometer constructed by core-diameter-mismatch welding. J. Appl. Opt..

[50] Tang C.P., Deng M., Zhu T., Rao Y.J. (2011). Photonic crystal fiber based M-Z interferometer for refractive index measurement. J. Optoelectron. Laser.

