# Supplementary material for: Design of Refractive Index Sensors Based on Valley Photonic Crystal Mach–Zehnder Interferometer
Source: Sensors (Basel). 2025 May 23;25(11):3289. doi: 10.3390/s25113289 (PMC12157726; doi:10.3390/s25113289)
Supplement: Supplementary file 1 [file sensors-25-03289-s001.zip › sensors-3641305-supplementary.pdf]

Supplementary Materials:

Design of refractive index sensors based on valley photonic crystal Mach–Zehnder interferometer

Yuru Li[1,2,†], Hongming Fei[1,2,3,†,*], Xin Liu[1,2] and Han Lin[4]

[1]*College of Physics and Optoelectronics, Taiyuan University of Technology, Taiyuan 030024, China*

[2]*Shanxi Key Laboratory of Precision Measurement Physics, Taiyuan University of Technology, Taiyuan 030024, China*

[3]*State Key Laboratory of Quantum Optics and Quantum Optics Devices, Shanxi University, Taiyuan, 030006, China*

[4]*Centre for Atomaterials and Nanomanufacturing, School of Science, RMIT University, Melbourne, Victoria 3000, Australia*

[*]*Correspondence: feihongming@tyut.edu.cn*

[†]*These authors contributed equally to this work.*

S1. Design of Mach–Zehnder interferometer based on valley photonic crystal

In order to design the refractive index sensor, we initially constructed a honeycomb lattice, as depicted in Figure S1(a). This structure consists of a silicon substrate and circular air holes. The thickness of the silicon substrate is h=220 nm. The lattice constant is set to a=440 nm. Within this configuration, each unit cell comprises two identical circular air holes with radii of RA=RB=80 nm, resulting in C6V rotational symmetry. The band structure of transverse electric (TE) field modes calculated by FDTD software reveals the presence of a Dirac point at the K(K′) valley of the VPC.

To achieve a photonic bandgap, we increased the radius of hole A to 120 nm and decreased that of hole B to 40 nm to create valley photonic crystal VPC1, and VPC2 is the mirror image of VPC1, as illustrated in Figure S1 (b). In this way, the K and K’ points degenerate to form a photonic bandgap of 271.89 nm (1373.47-1645.36 nm), as shown in Figure S1(c). As a result, C6V symmetry is reduced to C3 symmetry.

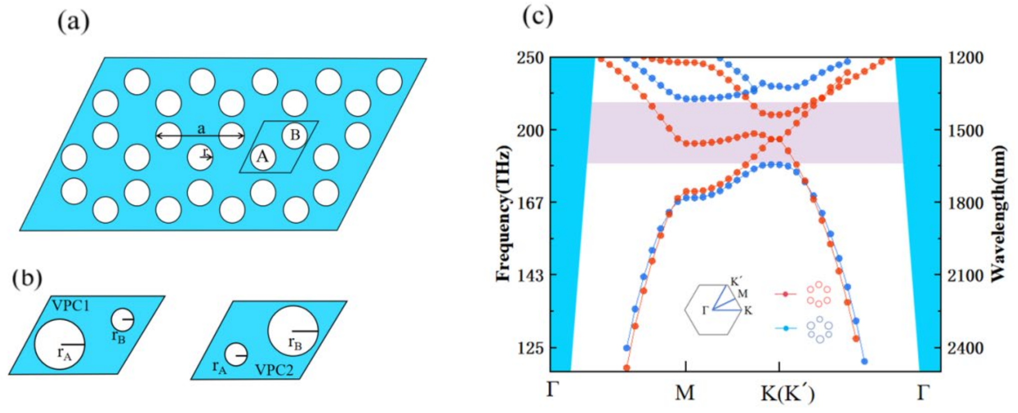

Figure S1. A schematic of the photonic crystal and photonic band diagram. (a) A schematic of the initial honeycomb photonic crystal structure. The parallelogram represents the unicell of two circular holes (A and B), where the lattice constant a=440 nm and the circle radius r=80 nm. (b) Two unicells of VPC1(left) and VPC2(right). (c) Photonic band diagrams of the original PC (red dot lines) and VPC1 (blue dot lines). The purple shading region marks the bandgap, and the blue shadow marks the air cone.

S2. Topological waveguide analysis with different boundary types

According to bulk boundary correspondence, topological phase transitions occur between different topological numbers at the interface. VPC1 exhibits Chern numbers of $C_{K'}$= -1/2 and C_K = +1/2 at the K' and K valleys, resulting in a total Chern number C=C_K+$C_{K'}$=0. Consequently, we introduced a new non-zero topological invariant called the valley Chern number, $C_V = (C_K - C_{K'})$ =-1. For VPC2, the Chern numbers at K' and K valleys are $C_{K'}$= 1/2 and C_K = -1/2, respectively. Thus, the valley Chern number of VPC2 is 1. The valley Chern numbers with opposite signs are essential for generating topological edge states at their boundaries. When constructing waveguides with VPC1 and VPC2, two combinations, namely zigzag-type and beard-type boundaries, are used to support topological edge states, as shown in Figure S2 (a) and (b). Transmittance spectra of straight waveguides composed of zigzag-type and beard-type boundaries are depicted in Figure S2 (c) and (d), respectively. The purple region represents the working bandwidth for both types of straight waveguides with different boundaries. It can be observed that the zigzag-type waveguide has a broader bandwidth and higher transmittance compared to the beard-type waveguide. Hence, we chose the zigzag-type waveguide to construct the MZI sensors in this study.

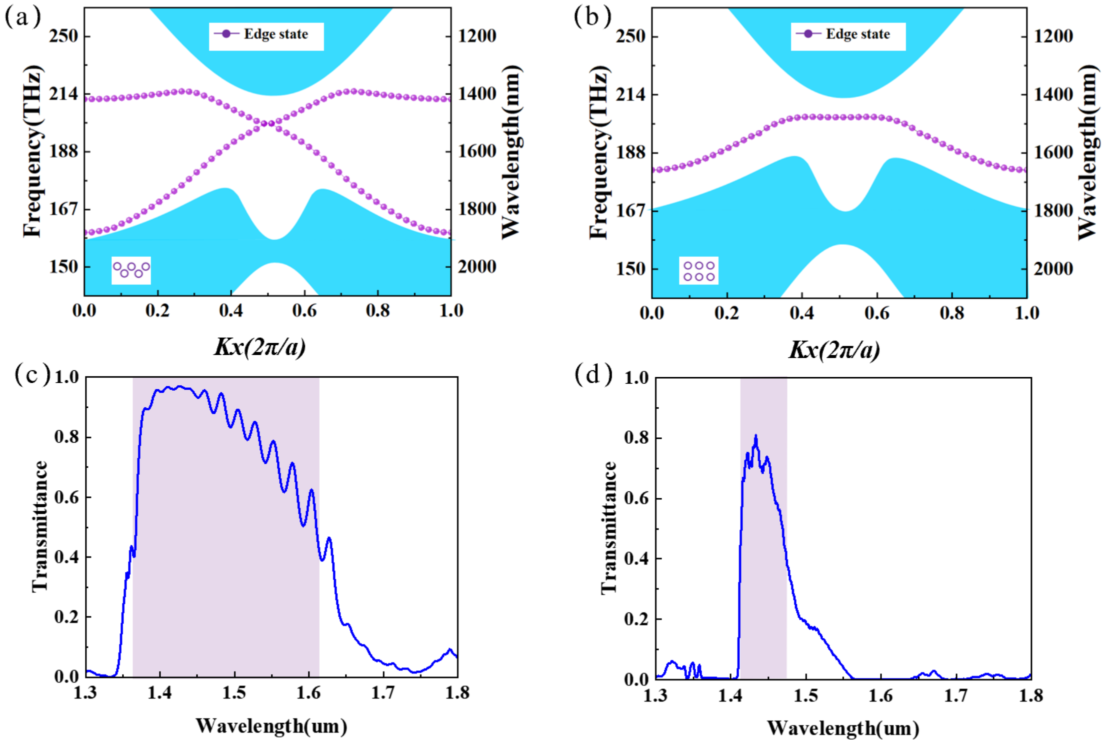

Figure S2. (a) An edge state band diagram of the zigzag-type boundary composed of VPC1 and VPC2. (b) An edge state band diagram of the beard-type boundary composed of VPC1 and VPC2. (c) The transmittance spectrum of the straight waveguide based on the zigzag-type boundary. (d) The transmittance spectrum of the straight waveguide based on the beard-type boundary.

S3. Influence of different refractive indices on edge states

The impact of varying refractive indices on the edge states of the zigzag-type boundary was further investigated. The edge state curves obtained with different refractive indices are depicted in Figure S3 and exhibit a redshift with increasing refractive index.

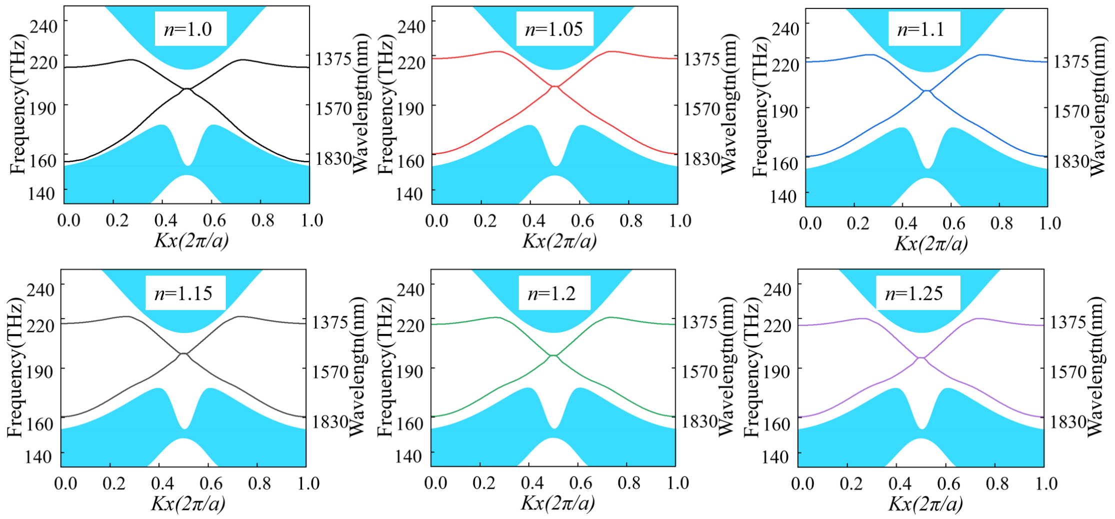

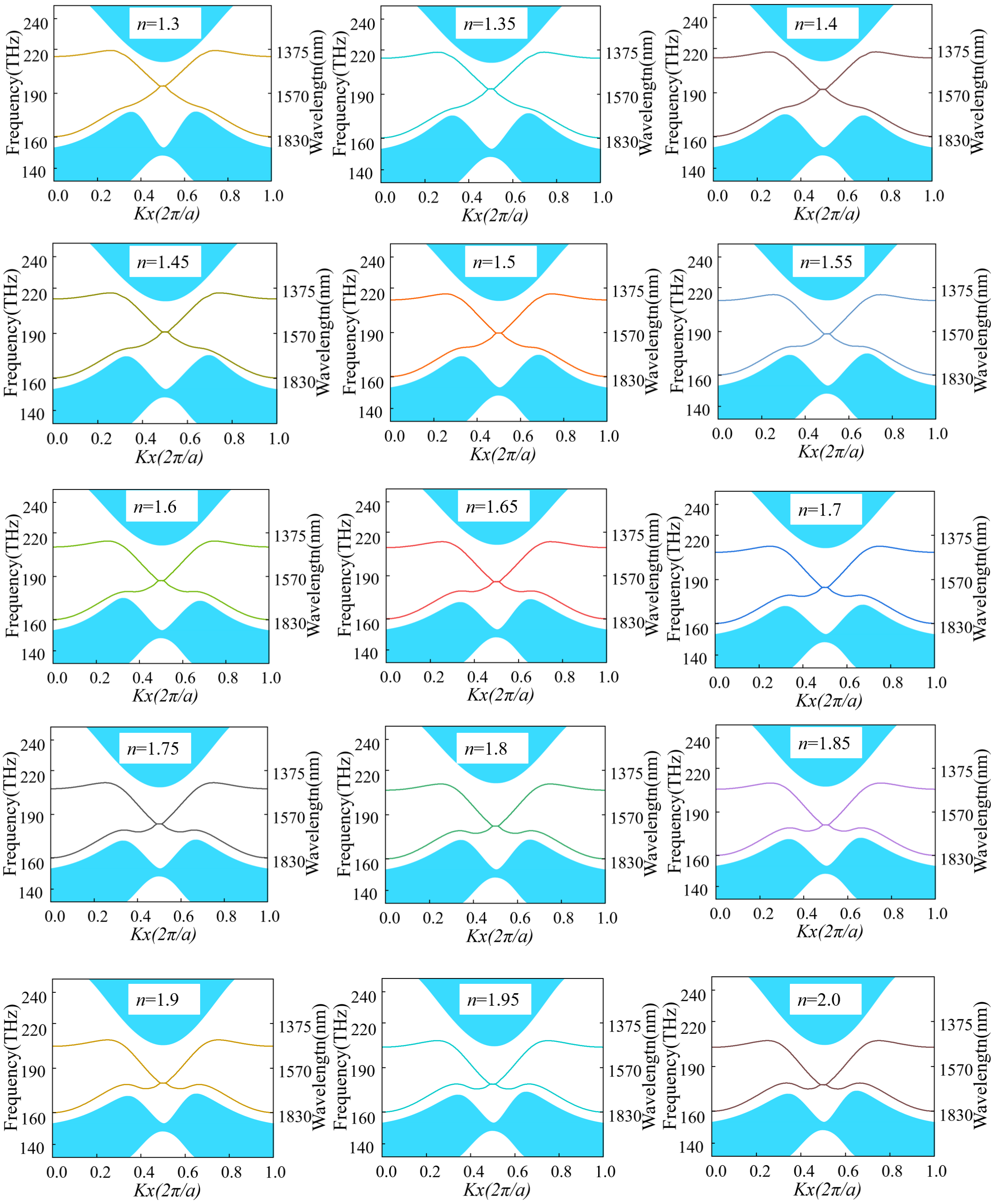

Figure S3. Photonic band diagrams of edge states of zigzag-type boundaries with different refractive indices.

S4. Straight waveguide at different sensing refractive indices

The topological straight-shaped waveguide structure based on the zigzag-type boundary is shown in Figure S4. The transmittance spectra of the waveguides with different refractive indices filled in the sensing part are shown in Fig. S5. The transmittance working bandwidth redshifts with the increased refractive index in the sensing part marked by the green shadow.

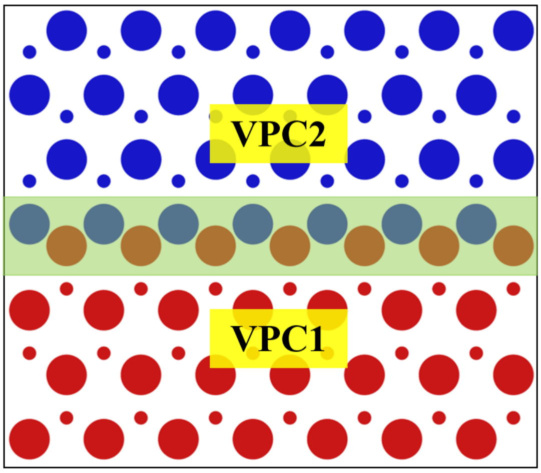

Figure S4. Schematic diagram of topological straight waveguide.

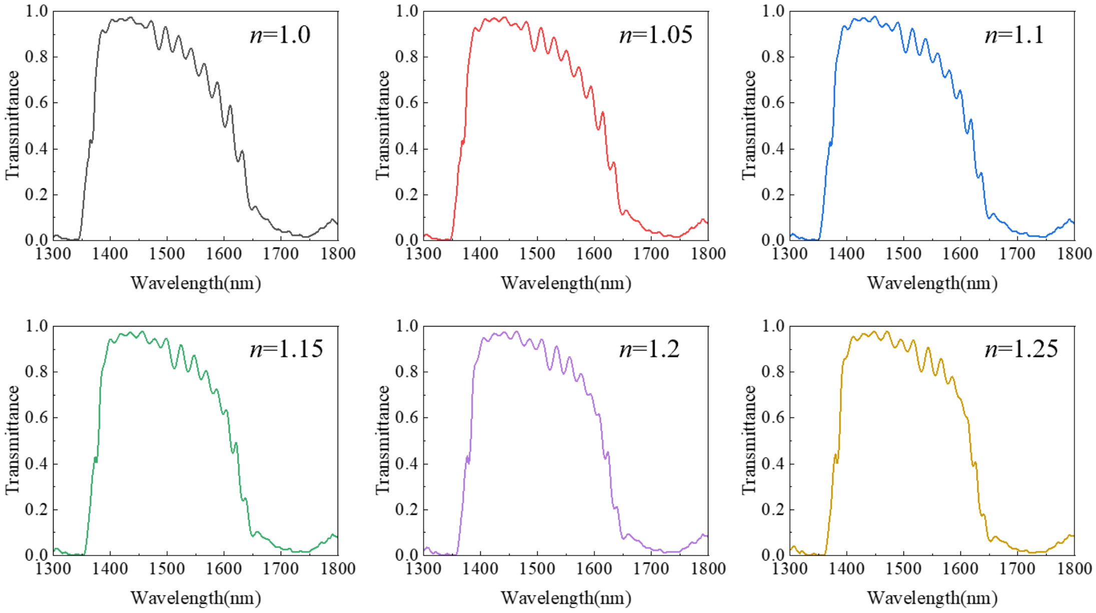

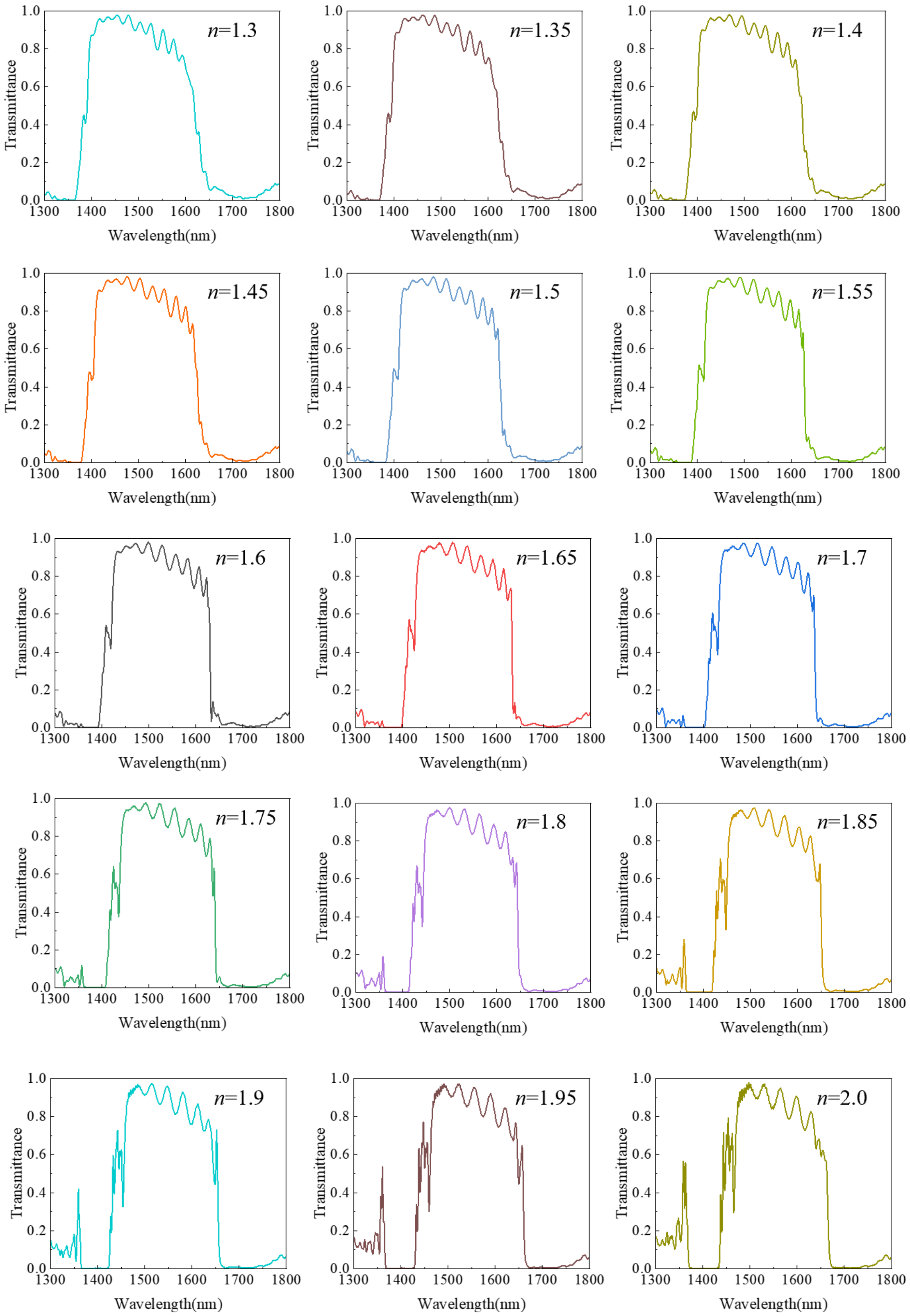

Figure S5. The transmittance spectra of straight waveguides with different refractive indices.

S5. Omega (Ω)-shaped waveguide at different sensing refractive indices

The topological Ω-shaped waveguide structure based on the zigzag boundary is shown in Figure S6. The transmittance spectra of the waveguides with different refractive indices filled in the sensing part are shown in Figure S7. The transmittance working bandwidth redshifts with the increased refractive index in the sensing part marked by the green shadow.

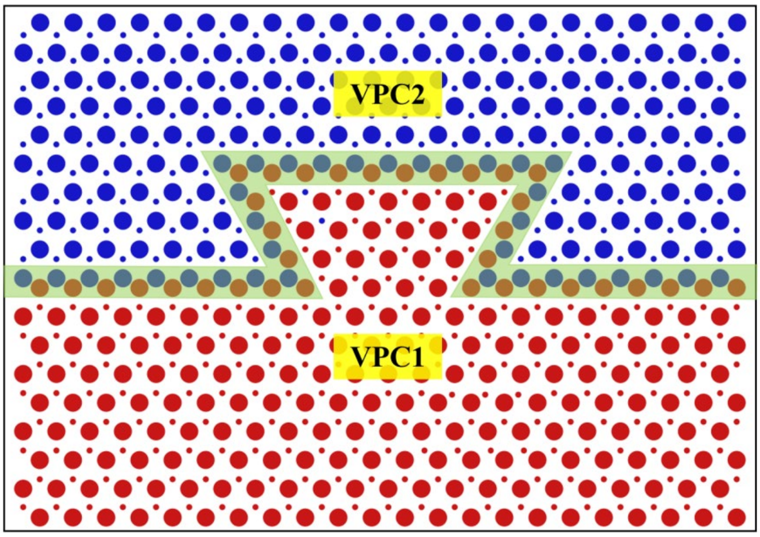

Figure S6. Schematic diagram of topological Ω-shaped waveguide.

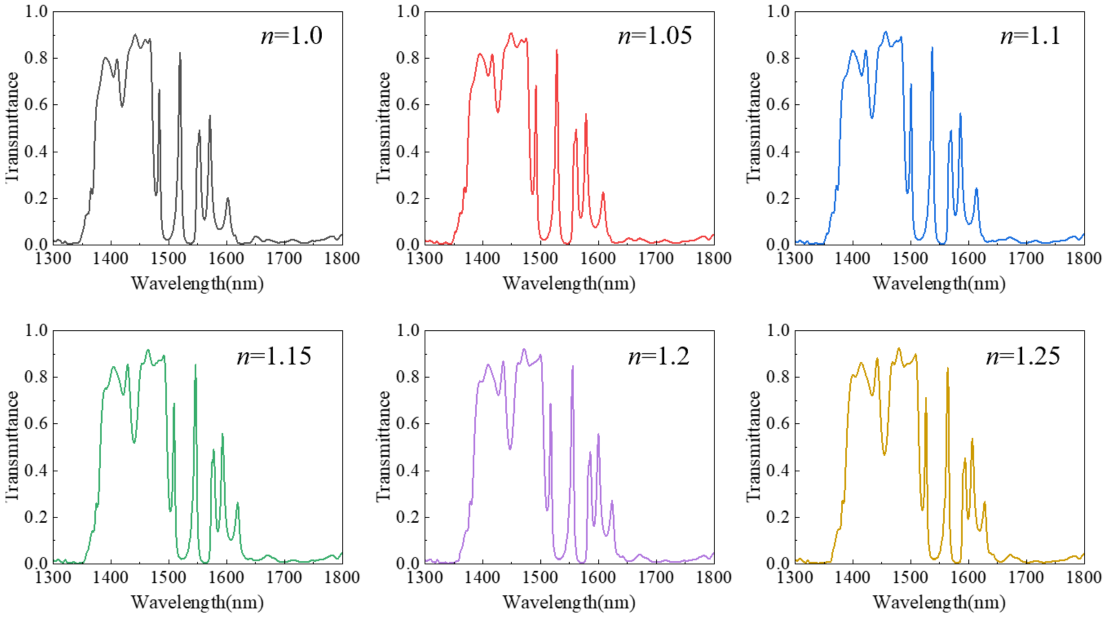

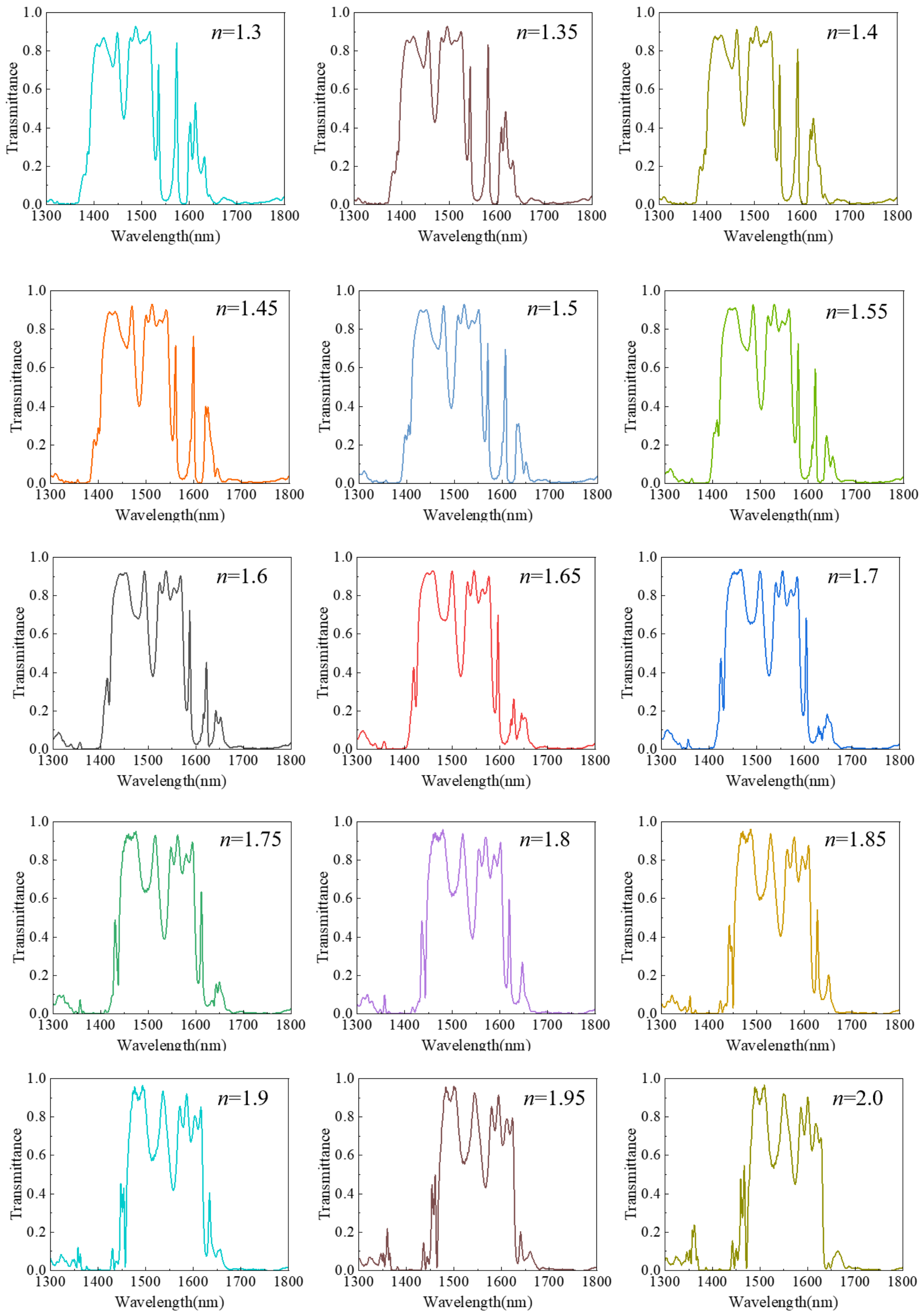

Figure S7. The transmittance spectra of Ω-shaped waveguides with different refractive indices.

S6. Topological waveguide analysis consisting of small holes

The edge state of the zigzag-type boundary of the topological waveguide composed of small holes is shown in Figure S8(a), and the transmittance is shown in Figure S8(b). The purple area represents the working bandwidth, and it can be observed that in the working bandwidth range, its transmittance is relatively low, so most of the light enters the two arms of the MZI, thereby reducing the loss as much as possible.

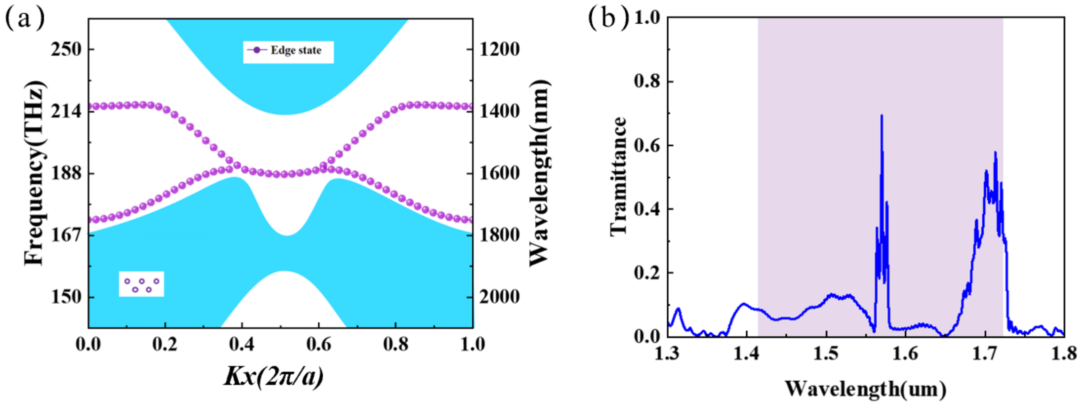

Figure S8. (a) An edge state band diagram of the zigzag-type boundary composed of VPC1 and VPC2 (small holes). (b) The transmittance spectrum of the straight waveguide based on zigzag-type boundaries composed of small holes.

S7. Transmittance spectra of MZI refractive index sensors with different refractive indices

We further investigated the sensing capability of MZI refractive index sensors with various path length differences of ΔL=2a, 5a, 10a, and 16a. The transmittance spectra are shown in the following figures.

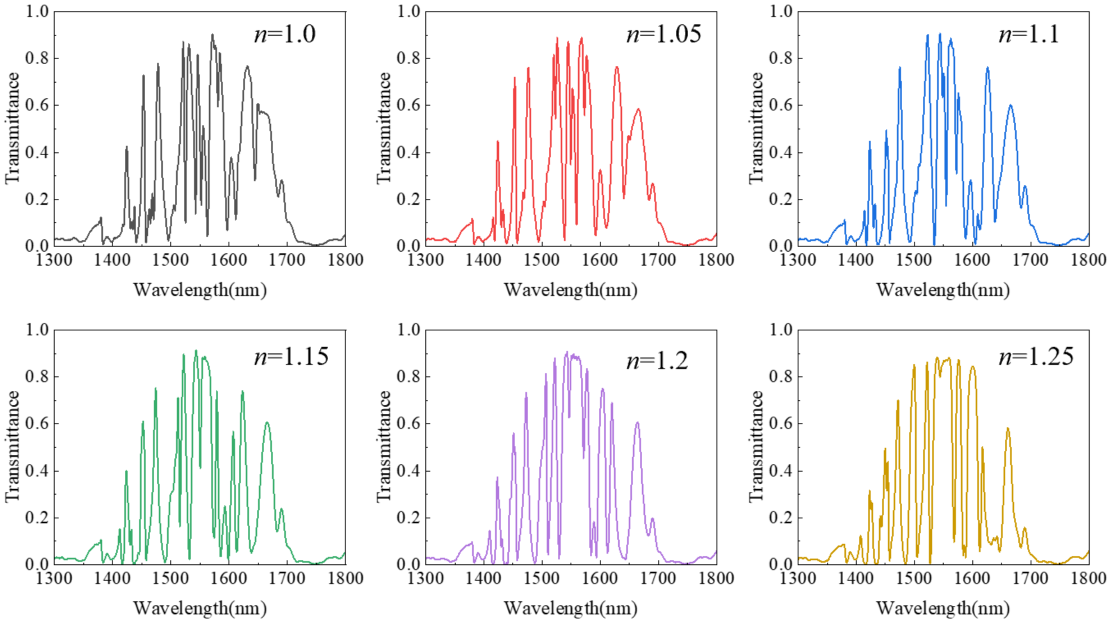
n=1.0
n=1.05
n=1.1
n=1.15
n=1.2
n=1.25
Transmittance
Wavelength(nm)

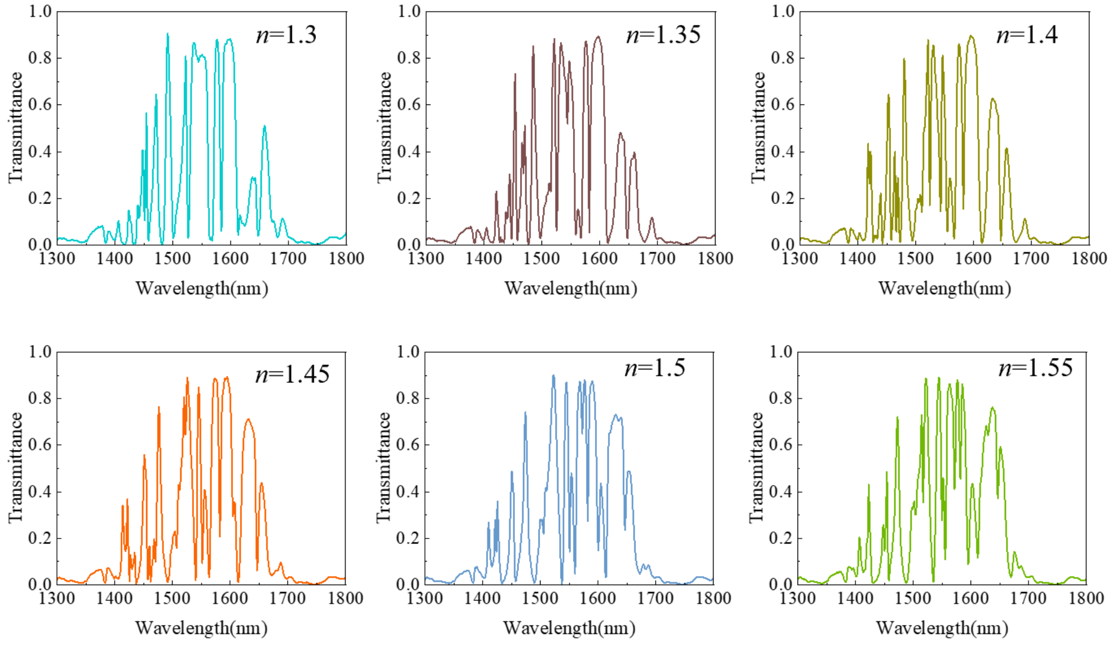
n=1.3
n=1.35
n=1.4
n=1.45
n=1.5
n=1.55
Transmittance
Wavelength(nm)

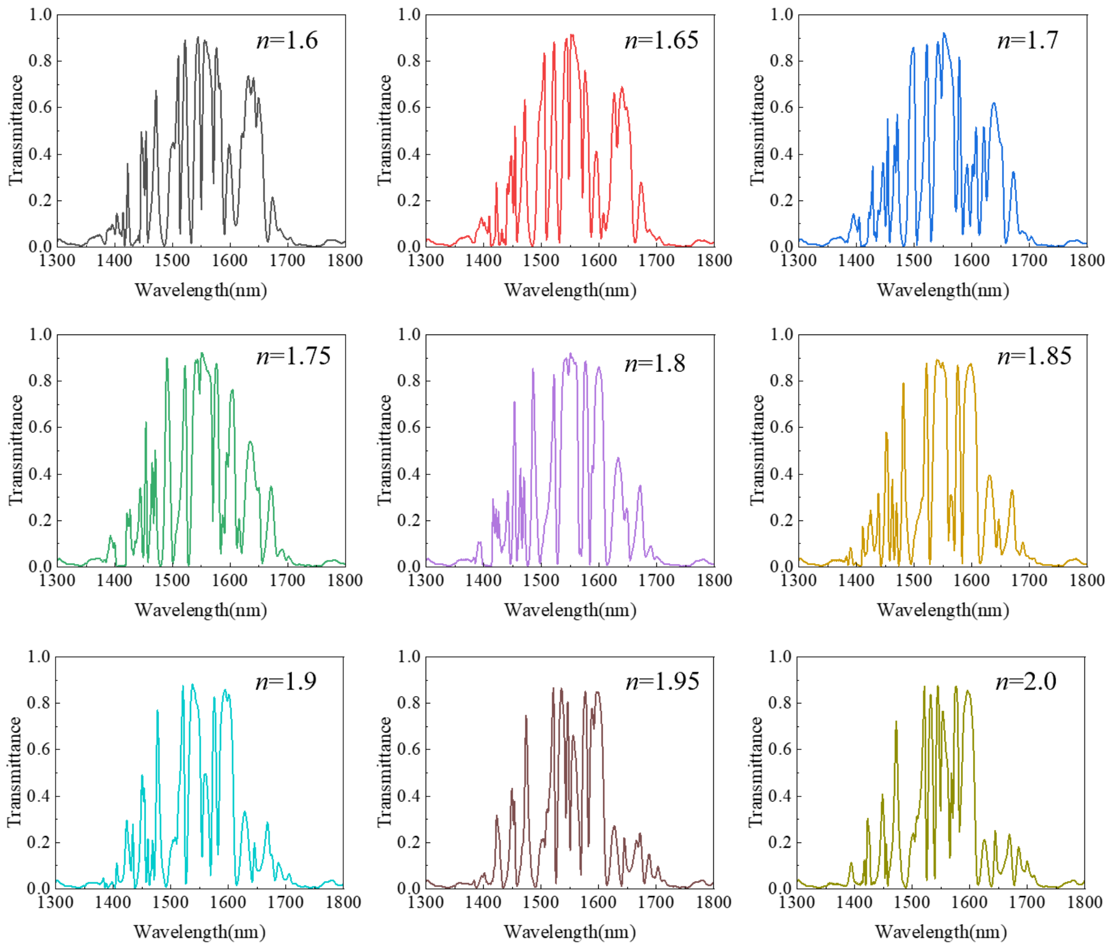

Figure S9. Transmittance spectra of MZI with different refractive indices when ΔL is $2a$.

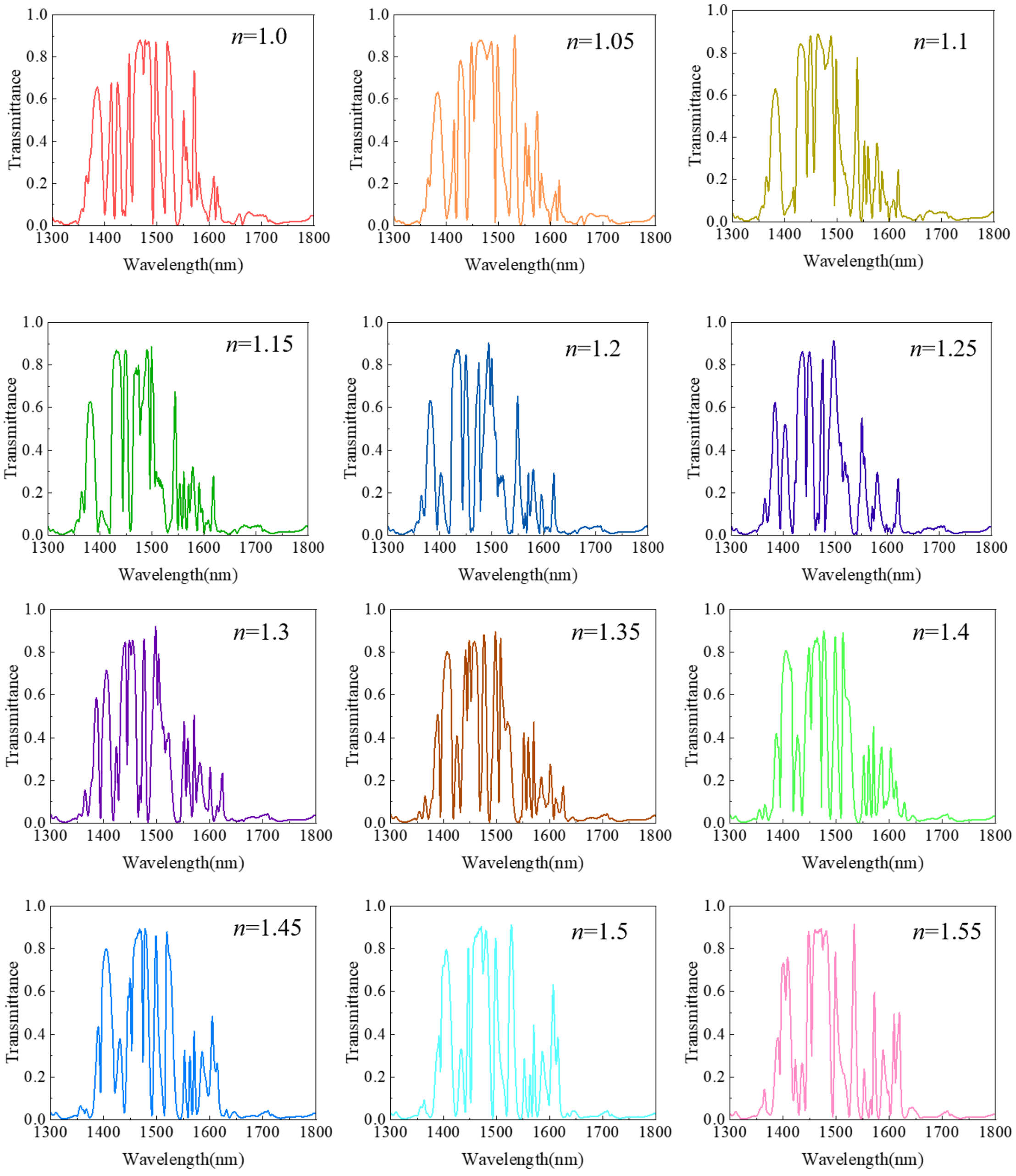

n=1.0
n=1.05
n=1.1
n=1.15
n=1.2
n=1.25
n=1.3
n=1.35
n=1.4
n=1.45
n=1.5
n=1.55
Transmittance
Wavelength(nm)

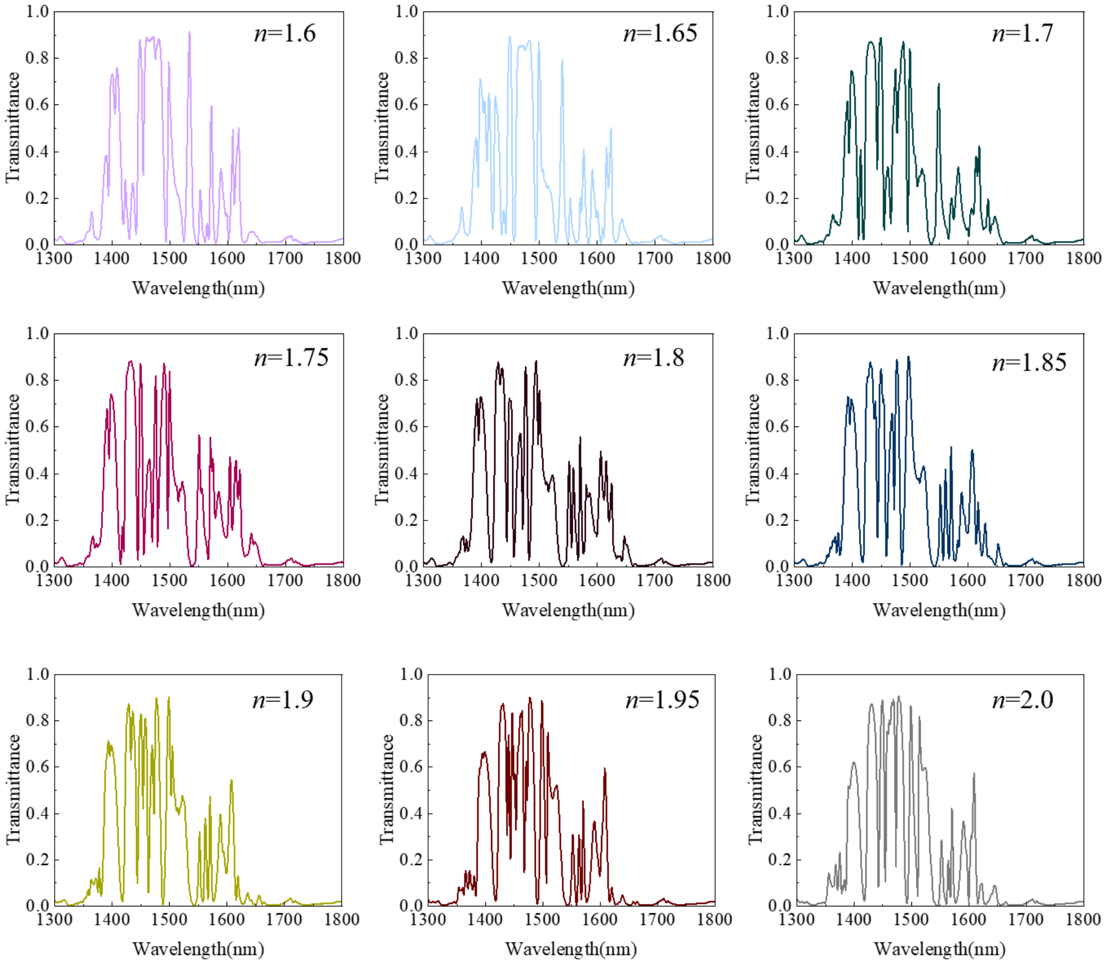

Figure S10. Transmittance spectra of MZI with different refractive indices when *ΔL* is 5*a*.

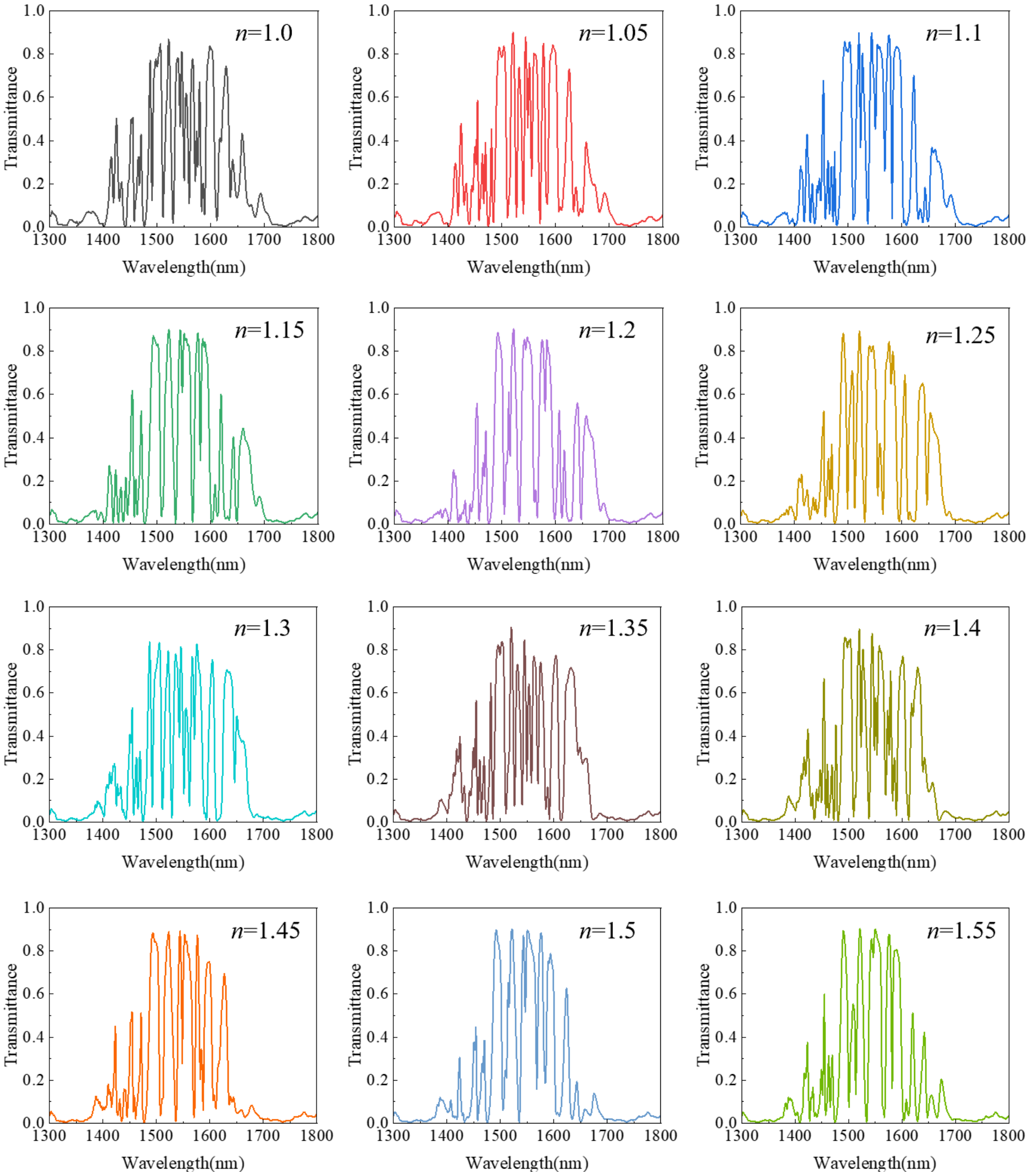
n=1.0
n=1.05
n=1.1
n=1.15
n=1.2
n=1.25
n=1.3
n=1.35
n=1.4
n=1.45
n=1.5
n=1.55
Transmittance
Wavelength(nm)
0.0
0.2
0.4
0.6
0.8
1.0
1300
1400
1500
1600
1700
1800

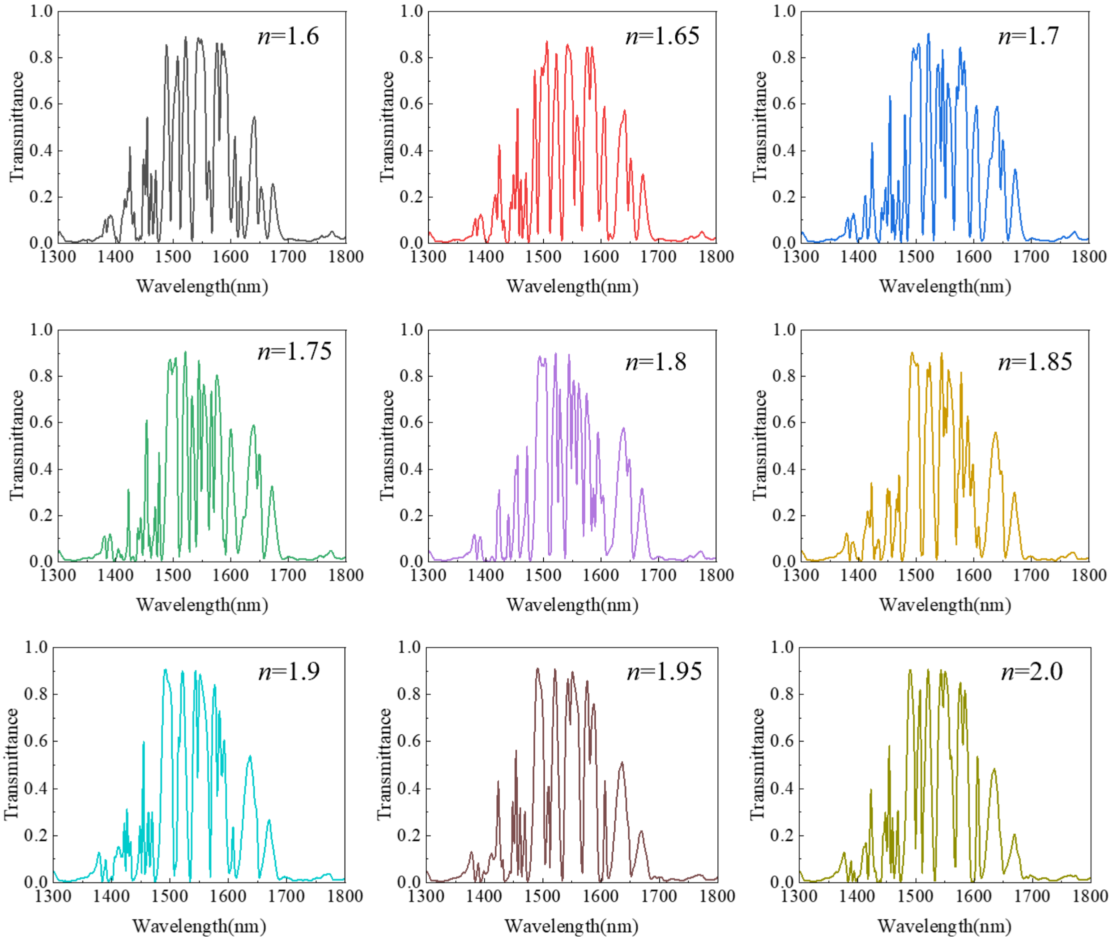

Figure S11. Transmittance spectra of MZI with different refractive indices when ΔL is $10a$.

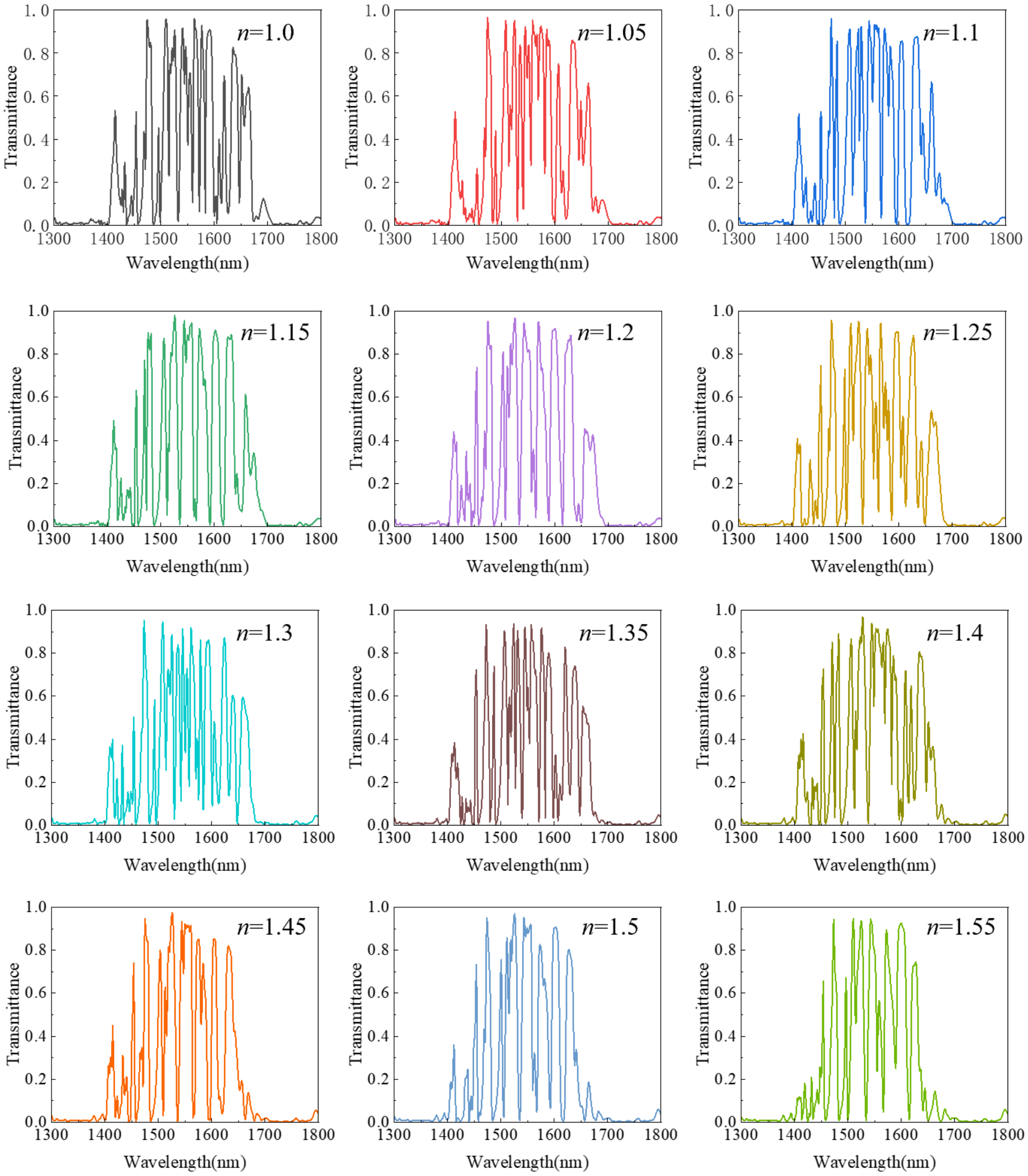

n=1.0
n=1.05
n=1.1
n=1.15
n=1.2
n=1.25
n=1.3
n=1.35
n=1.4
n=1.45
n=1.5
n=1.55
Transmittance
Wavelength(nm)

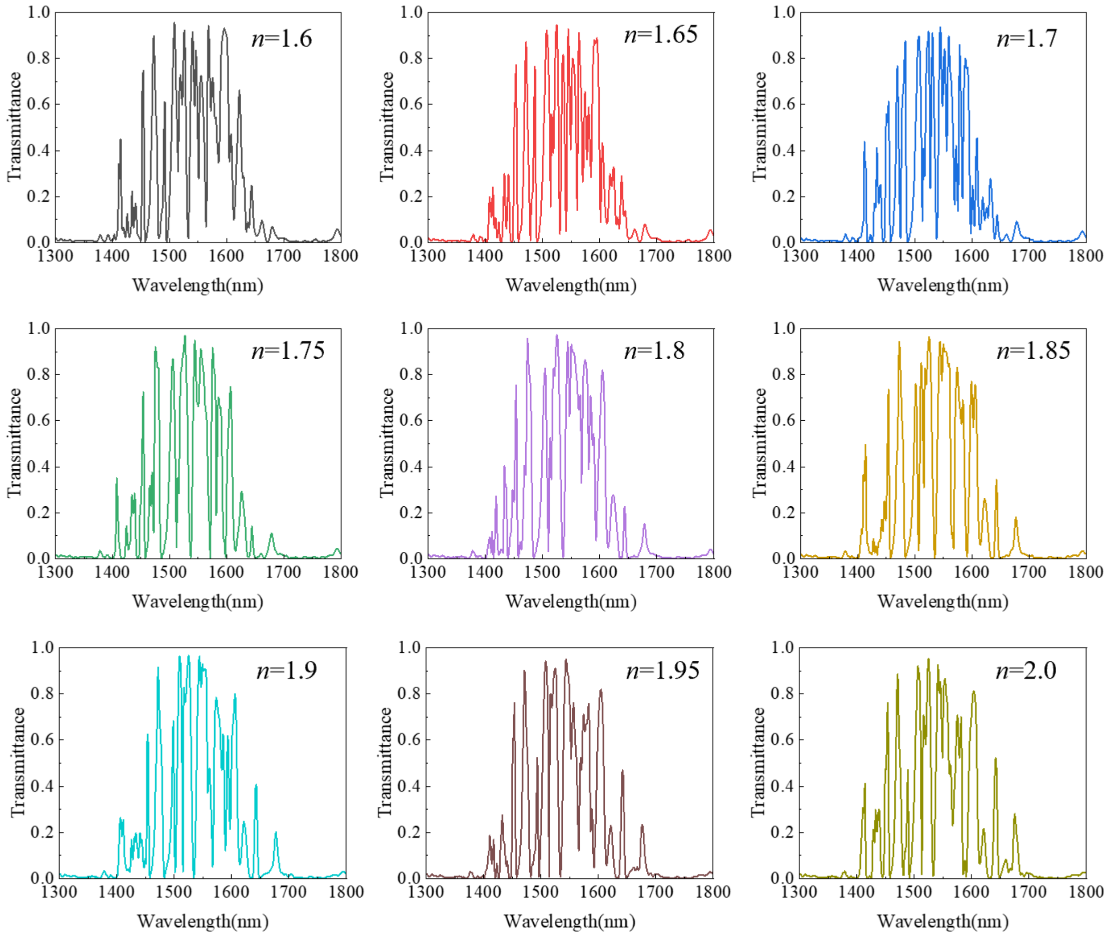

Figure S12. Transmittance spectra of MZI with different refractive indices when ΔL is 16a.

S8. The response of the sensor to environmental temperature fluctuations

In addition, we discussed the impact of environmental disturbances, such as temperature, on the sensor. When ΔL is 5a, the transmittance spectrum of the VPC MZI structure under temperature variations from 293 K to 300 K is shown in Figure S13 (a) and (b). The temperature variation from 293 K to 300 K resulted in a deviation in the transmittance spectra of 1.3%. The result demonstrates that small temperature perturbations exhibit negligible impact on the transmission characteristics, highlighting the robust thermal stability of the proposed topological photonic configuration.

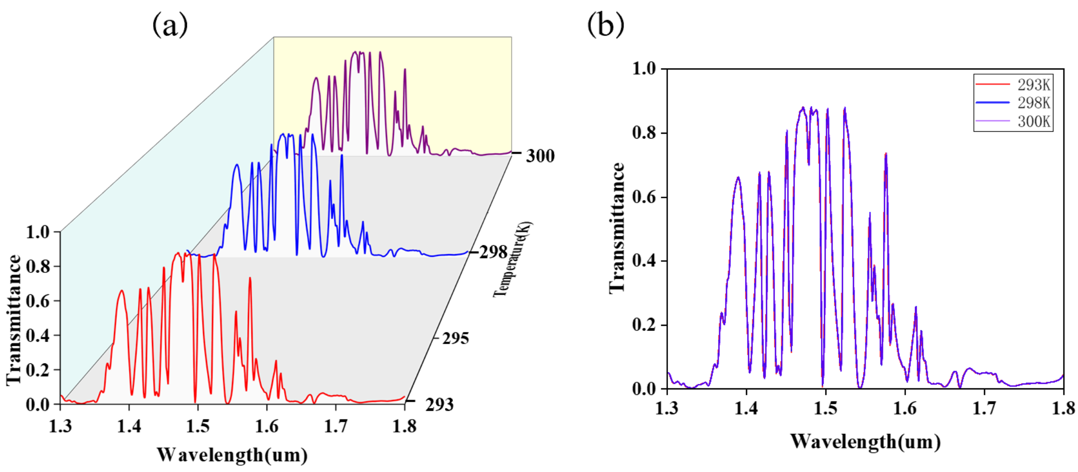

Figure S13. (a) Transmittance spectra of MZI at different environmental temperatures. (b) Comparison of transmittance spectra of MZI at different environmental temperatures.

S9. The CMOS compatibility statement and specific process steps

The valley photonic crystal slab can be fabricated on a standard silicon-on-insulator (SOI) wafer with a 250 nm thick top silicon layer and a 3 µm- thick buried oxide layer using a standard CMOS fabrication technique. The fabrication process can be carried out in two steps: (i) patterning of the top Si layer using electron beam (EB) lithography followed by reactive ion etching (RIE) (shown schematically in Figure S14 (a)); (ii) selective removal of SiO_2 under the PC structure by wet etching using a patterned photoresist as a mask (shown schematically in Figure S14 (b)).

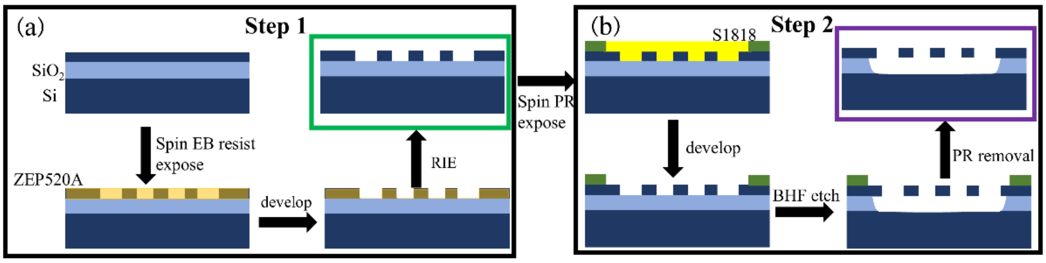

Figure S14. The fabrication procedure of the sample containing the topological photonic crystal slab. (a) Step 1: The top silicon layer is patterned using standard electron beam (EB) lithography, followed by reactive ion etching (RIE). (b) Step 2: The silicon dioxide below the photonic crystal is selectively etched away using wet etching with a patterned photoresist (PR) as a mask.

S10. The fabrication tolerances for the hole radius and lattice constant in the

VPC design

In addition, we further calculated the transmittance spectrum of the refractive index sensor with a random distribution of the hole radius *r* (±5%) and lattice constant *a* (±5%) in the VPC design, as shown in Figure S15. Through data analysis, by only changing the air hole radius *r* (±5%), the deviation in the transmittance curve is 0.9%. In comparison, variation in the lattice constant *a* (±5%) results in a deviation of 1.7% in the resulting transmittance curve. When both r and a are changed simultaneously, the deviation in the transmittance curve is 4.8%. From Figure S15, it is evident that the transmittance spectrum has remained largely unchanged. Therefore, the variations in the hole radius and lattice constant caused by the VPC manufacturing process do not impact the device's performance.

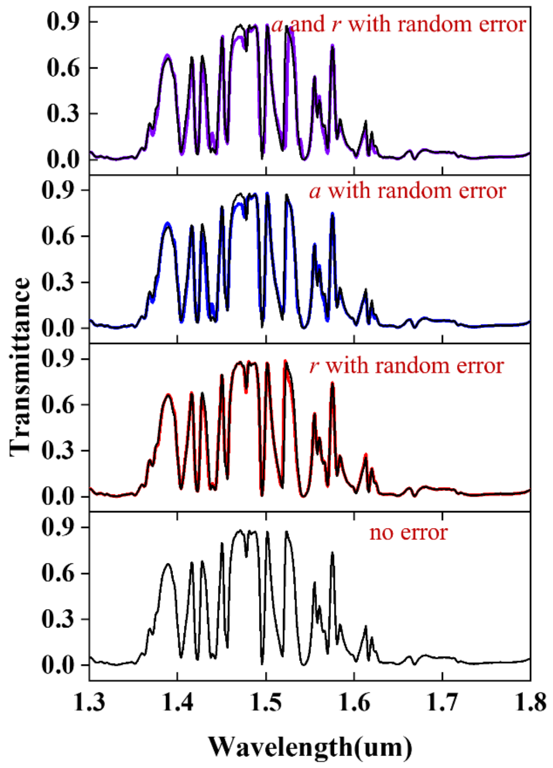

Figure S15. Transmittance spectra of MZI with manufacturing errors in hole radius and lattice constant, and comparison with MZI transmittance curve without errors.

S11. The potential sources of experimental error in transmittance measurements.

The main factor that will affect the experimental measurement of the transmittance spectrum is the coupling efficiency of the entire system. In the experiment, light should

be coupled to the MZI refractive index sensor using optical fibers and diffraction gratings. The coupling efficiency strongly depends on the incident angle of the light from the optical fiber and the distance between the fiber facet and the grating, which may vary significantly, making it hard to measure the transmittance accurately. This error can be minimized by using a straight ridge waveguide as a reference, as demonstrated in previous work [1]. In this way, the overall transmittance and the shift in the interference peaks and valleys can be accurately measured through comparison with a straight waveguide. The measured transmittance spectrum will be normalized using the transmittance spectrum of the straight waveguide to remove the errors caused by the coupling efficiency. In addition, the fluctuation in the environmental temperature may also affect the measurement, which can be minimized by putting the samples in a chamber with a constant temperature.

Reference

1. Shalaev, M. I.; Walasik, W.; Tsukernik, A.; Xu, Y.; Litchinitser, N. M., Robust topologically protected transport in photonic crystals at telecommunication wavelengths. *Nat. Nanotechnol.* **2019,** *14*, 31-34.
